# Integrated Portable and Stationary Health Impact-Monitoring System for Firefighters

**DOI:** 10.3390/s24072273

**Published:** 2024-04-02

**Authors:** Panagiotis Lioliopoulos, Panagiotis Oikonomou, Georgios Boulougaris, Kostas Kolomvatsos

**Affiliations:** Department of Informatics and Telecommunications, University of Thessaly, 3rd km Lamia–Athens, 35100 Lamia, Greece; plioliopoulos@uth.gr (P.L.); gboulougar@uth.gr (G.B.); kostasks@uth.gr (K.K.)

**Keywords:** air quality monitoring, health impact, fire management, emergency management, restful API

## Abstract

The multi-layered negative effects caused by pollutants released into the atmosphere as a result of fires served as the stimulus for the development of a system that protects the health of firefighters operating in the affected area. A collaborative network comprising mobile and stationary Internet of Things (IoT) devices that are furnished with gas sensors, along with a remote server, constructs a resilient framework that monitors the concentrations of harmful emissions, characterizes the ambient air quality of the vicinity where the fire transpires, adopting European Air Quality levels, and communicates the outcomes via suitable applications (RESTful APIs and visualizations) to the stakeholders responsible for fire management decision making. Different experimental evaluations adopting separate contexts illustrate the operation of the infrastructure.

## 1. Introduction

Wildfires are one of the most common natural disasters in many regions, most commonly across the Mediterranean Sea and areas in the taiga biome. The profound impact of wildfires is evident not only in the destruction of landscapes but also in the significant health risks posed to the wildland firefighters engaged in extinguishing these fires. Large amounts of gaseous emissions and particulate matter are emitted from fire and smoke due to biomass burning. Wildland firefighters are exposed to high concentrations of these pollutants for an extended amount of hours, resulting in a severe and immediate health impact. According to [1], there are acute health effects from smoke exposure, mostly involving lung functioning, pathological issues, as well as long-term effects, such as cardiovascular diseases and lung cancer. The research results presented in [2] show that prolonged exposure to wildfire smoke increases premature mortality, respiratory issues and increased population cancer risk, with a higher occurrence of lung cancer and wider respiratory complications.These problems manifest themselves immediately, usually from the first time the firefighters are exposed to wildfire. When a wildfire occurs, firefighters are usually under constant toxic smoke exposure for many hours, lacking adequate protection equipment since there are none that meet the specification of relevant associations, forcing them to resort to protection solutions, such as bandanas or heat-resistant shrouds, which offer limited or no pollutant protection. According to [3], these solutions have been proven to be ineffective for total particulate matter filtration. In [4], the authors state that direct evaluations of the performance of respirators for wildland firefighters are disjointed since the use of different respiratory protection methods is impractical for several reasons, such as short-term protection against long-term occupation, the weight of the equipment and the reduced mobility of the equipment, suggesting that more prescribed burn studies be conducted as well as researching more effective respiratory protection methods.Besides serious health concerns, the research in [5] measured the blood pressure and heart conditions of the wildland firefighters the day before, after and the morning following prescribed fires, resulting in a reduction in systolic blood pressure and increases in heart rate immediately after and the following morning, most likely resulting from multiple fireline factors, such as exposure to fire and smoke, the temperature and the tasks.

The composition of the smoke from wildfires further compounds the health risks faced by firefighters. Studies by [1,6] reveal that the smoke contains multiple pollutants resulting from biomass burning. These pollutants manifest in gases and particulate matter forms. They are composed of mainly carbon content, most of it being carbon dioxide (CO_2_) and carbon monoxide (CO), particulate matter of an aerodynamic diameter of less than 2.5 μm^3^ (PM_2.5_), and inorganic gases, hydrocarbons, oxygenated hydrocarbons, trace metals and other particulate matter of different diameters. Complex compounds were also identified, such as formaldehyde and benzene [6]. Inhaling quantities that exceed a specified limit leads to serious health issues as described above. Most of the time, firefighters are exposed, substantially inhaling these emissions. Under these conditions, wildland firefighters stay out of the smoke when they can and try to breath clean air, whenever possible. Of course, such movement is perceived by firefighters using their senses. Hence, the perception of pollutants in the air is significantly reduced, potentially leading to decisions that still do not prevent wildland firefighters from inhaling emissions. It can easily be understood that the point of interest is to avoid exposure in large quantities of smoke and fire emissions.

The deflection of exposure to pollutants can be achieved by the constant knowledge of air quality in the place of interest. In order to maintain constant knowledge, the utilization of monitoring techniques is necessary. Modern monitoring is achieved through Internet of Things (IoT). Internet of Things integrates multiple technologies, such as communication protocols, wireless technologies, edge computing, cloud computing, security technologies, embedded systems and machine learning and artificial intelligence. The integration of these technologies permits IoT to be applied to a multitude of fields, such as health, environment, agriculture, industry and household. The fundamental concept of the IoT is the connection between multiple objects over IP networks. These objects are capable of communication, sensing and, nowadays, intelligence. Such capabilities enable the constant observation of air quality and the distribution of these data through the Internet in real time, achieving real-time monitoring and decision making.

In this paper, we present a monitoring IoT system for detecting and recording the air quality, pollutants of the fire and smoke from wildfires for firefighters. The functionalities of this system revolve around the real-time observation of pollutant values, constant data transmission to the headquarters and data storage and manipulation. The observation of pollutants is realized through a wireless network, containing multiple sensor systems communicating with a remote server. The server is responsible for data storing and manipulation and real-time visualization of the pollutant values and the quality of the air. The observation component collects information regarding the pollutants as well as geospatial information. This system can benefit wildland firefighters in many ways. First and foremost, they are provided with real-time precise knowledge of the quality of the air on their location. Hence, this knowledge can assist in decision making, resolving the safe distancing from the smoke and fire. The benefits of this is the health safety of the wildland firefighters as well as maintaining strength during fire extinguishing. Aside from direct benefits to firefighters, the data collected can be used to predict the behavior of wildfires. Thus, more efficient planning and resource allocation can be achieved.

The structure of the remaining paper is as follows. Section 2 describes the relevant related work. Section 3 describes the architecture of the sensors and the server. Section 4 describes the applied technologies for the development as well as the REST API interface for communication between the server and the portable solutions and the stationary solutions. Section 5 presents the experimental evaluation of the portable solution under different fire combustion conditions. Finally, Section 6 concludes the findings of this work, providing future work suggestions on top of this work.

## 2. Related Work

Monitoring firefighters has been thoroughly studied and because of the technological advancements in the hardware, many applications have been implemented for monitoring them during fire suppression, both in outdoor and indoor environments. Several studies have monitored physiological and physical parameters, as well as the safety of firefighters. However, monitoring air quality in a wildfire environment has hardly been examined. This is because the main interest is shifted in the situation of the firefighters at the time of fire suppression. Such systems utilize a set of sensors responsible for measuring these parameters, as well as a processing unit, and they usually operate under a network, between the firefighter and the headquarters. Their features in their designs differentiate along several axes. The location where such systems are placed vary significantly. The most common approach is a wearable device, where the system is developed for the firefighters to wear it on their body. The location of the device can be the hands [7,8,9,10,11], the head [12] or in multiple locations on the body [13]. Wearable devices are the most common approach in these systems, as they offer multiple ways to implement the components of such systems. Subsequently, some systems have taken this idea one step further and have developed an intelligent suit [14,15,16,17,18,19], which integrates the sensor and processing unit inside the suit, resulting in a compacted system. Aside from wearable designs, some studies present non-wearable approaches [12], which limits the capability to acquire vitals from firefighters.

The physiological and physical parameters recorded from these devices vary for each system as well. Most studies include physiological parameters, such as heart rate, pulse rate, epidermal temperature and blood pressure [7,9,10,11,12,15,17,18,19], which are meant to indicate the status of the firefighter. For example, [15] developed an intelligent garment responsible for monitoring the physiological state of the firefighter through an electrocardiogram, electrodermal activity, an electromyogram, respiration, blood volume pulse, as well as from other physical parameters, specifically temperature and motion measurements through an accelerometer. Physical parameters, such as temperature, humidity and body movement detection through acceleration, are considered in the implementation of most systems [7,9,10,11,12,13,14,15,17,18,20]. Acquiring such values expands the capabilities of firefighters’ health monitoring since aside from physiological values, vital signs such as motion or posture can be extracted. Regarding air quality, a smaller number of studies join air quality monitoring with the rest of the system [7,11,17,18,20].

Similarly, many studies resolving air quality monitoring have been proposed; while they follow the same idea as the firefighter-monitoring devices, they are not worn (wearable device or intelligent garment). These applications measure pathogenic emissions from gasses [21], particulate matter [22] or both [23,24,25,26,27,28,29], where some focus on measuring the Air Quality Index (AQI) of the environment [21,22,24,25], although none of the latter provide any information about the wildfire environments.

The communication infrastructure varies as well, as it depends on the location of the monitoring system. A popular technology used is Wi-Fi, which is used for systems inside urban areas [20,21,22,26,29] or industrial areas [28], while for firefighter-monitoring systems, it is used for communication between a more powerful intermediate device (mobile phone [10] or a broker [7]), or capable processing units (such as Raspberry Pi). For longer ranges with less power consumption, technologies like LoRaWAN [9,12,13,23,25] and ZigBee/XBee [7,14] are utilized. When no remote server is required and the data are displayed locally, Bluetooth or other small-range technologies are used [8,11,15,16,17,18,19,24,27].

Table 1 and Table 2 demonstrate the aforementioned monitoring solutions accompanied by some aspects of their implementation.

## 3. System Architecture

**Figure 1:**
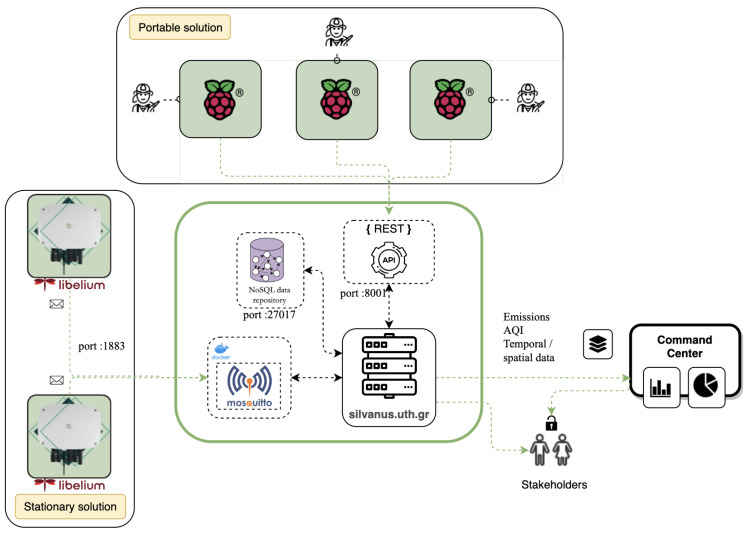
System architecture overview.

Figure 1 demonstrates an overview of the Health Impact Assessment system. This system is built upon three core components: (i) a portable and a (ii) stationary solution dedicated to monitoring air quality, particularly in the context of individuals exposed to a wildfire incident, alongside (iii) a web application managing the data flow from these solutions. The first component targets equipping frontline first-responders, particularly firefighters, with a compact node housing a Raspberry Pi, five emission sensors, and a communication unit. Data from these nodes are seamlessly transmitted and recorded in a MONGODB (https://www.mongodb.com/, accessed on 3 March 2024) database through PUT requests directed at specific endpoints. The second component, the stationary solution, comprises a set of Libelium Smart Spot models strategically positioned in safe areas near the wildfire incident. These devices are well suited for monitoring various environmental factors such as air quality, temperature, and humidity. Data from these devices are transferred via an (Message Queuing Telemetry Transport) MQTT (https://mqtt.org/, accessed on 3 March 2024) broker to interested parties. Finally, the web application acts as the third component, receiving and storing observations from both systems into a repository. Command centers and stakeholders interested in air quality and its impact on health can easily access the latest emission data, AQI and spatiotemporal information through straightforward GET requests to designated endpoints. Access to these resources requires authentication using credentials for effective utilization by our partners.

### 3.1. The Portable Solution

According to [30], the basic structure of each node consists of four basic units: the sensing/identification unit, the processing unit, the communication unit and the power supply unit. The processing unit is responsible for controlling the components of the sensor node and performs any required computations [30] as well as data preservation. The sensing/identification unit is the gateway to the physical world, i.e., through it, the node can understand physical phenomena and convert them to electrical signals. The communication unit manages the communications of the node with other components or remote server. Finally, the power supply unit supplies the system with energy and, usually, it consists of a battery and sometimes a power generator, such as solar panels.

The above units share some common parameters that must be taken under notice. Since this equipment is not fixed in position, rather being carried by personnel, the dimensions and the weight of each component should be taken under serious consideration because of the movement and stamina effect on the personnel. In addition, the temperatures and humidity under which they can function are of the utmost importance since the environment of interest contains extreme environmental conditions. Another important parameter is the lifespan. Although this seems trivial, a short lifespan, in addition to low tolerance to extreme temperature and humidity, means that the units must be replaced at regular intervals, increasing the cost of maintenance of each node. Finally, the accuracy of measurements of the sensing units must be sensitive enough in order to cover all the levels of the AQI.

**Figure 2:**
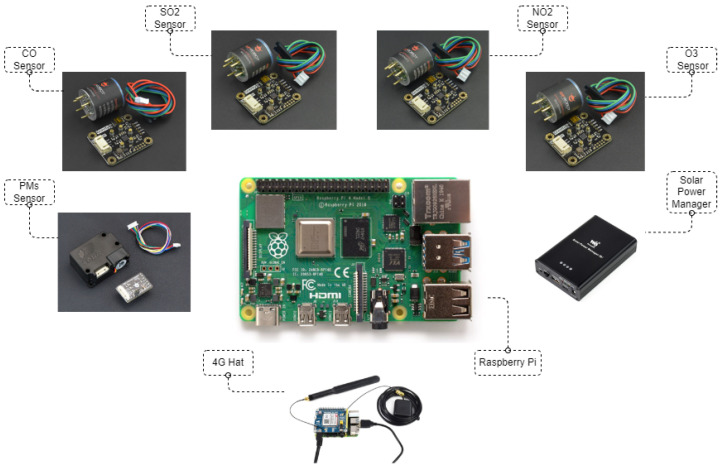
Portable solution: building blocks.

**Figure 3:**
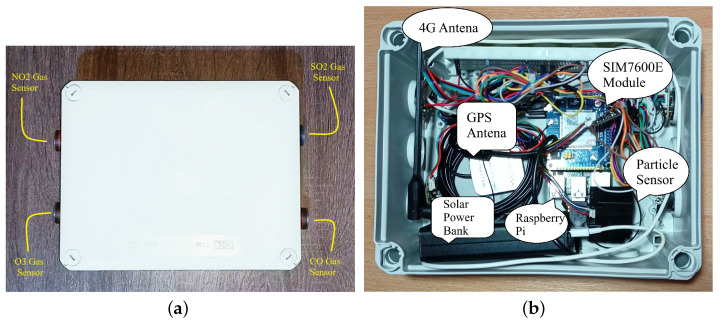
Complete portable air quality observation system. (**a**) Assembled node. (**b**) Interior System and Components.

In this work, we utilize the Raspberry Pi (https://www.raspberrypi.org/, accessed on 3 March 2024) microcomputer as the processing unit. On top of it, we attach the Waveshare SIM7600E HAT (https://www.waveshare.com/sim7600e-h-4g-hat.htm, accessed on 3 March 2024) extension as the communication unit that is also responsible for providing geospatial information about the location of the rescue personnel. The sensing unit consists of a set of sensors from DFRobot Gravity Series: four gas sensors (https://www.dfrobot.com/product-2516.html, accessed on 3 March 2024) and three particulate matter sensors (https://www.dfrobot.com/product-1272.html, accessed on 3 March 2024). Finally, for the power supply unit, we utilize the Waveshare Solar Power Management (B) (https://www.waveshare.com/solar-power-manager-b.htm, accessed on 3 March 2024) module, which operates both as a solar power energy transducer and as a battery since it can store energy. The building blocks of the proposed portable solution are demonstrated in detail in Figure 2. Figure 3a illustrates an assembled final node of the portable solution, while Figure 3b presents the inside of the sensor system and its components. The solution’s weight is less than 1.5 kg, and its dimensions are 25 cm × 20 cm × 15 cm, making it lightweight and compact.

The adoption of a fully functional mini-computer like Raspberry Pi in a highly demanding environment where adverse conditions prevail offers numerous advantages over conventional wearables. Beyond the implementation of simple repetitive tasks, such as monitoring the concentrations of fire emissions in our case offered by wearables and micro-controllers, the proposed solution can perform tasks that require more processing power and interactivity (presented in subsequent sections). It is also easier to expand its capabilities indicatively by adding other sensors (i.e., meteorological and biometrics) and/or providing new functionality like incorporating machine/federated learning algorithms to predict the expansion progress of the pollutants emitted by the fire occurrence [31]. Finally, this solution can be mounted on UGVs (Unmanned Ground Vehicles), UAVs (Unmanned Aerial Vehicles), and fire trucks, while it could be used in safe shelters as well.

As far as the drawbacks of the proposed solution are concerned, they are associated with the impact on the costs of communication and energy. The use of HTTP is expensive in communication resources, causing more overhead over the network, and requires more mobile data consumption compared to other communication protocols in the literature, despite the handy infrastructure for REST API. The system’s power consumption is greater due to the usage of Raspberry Pi, alongside 4 I2C (Inter-Integrated Circuit) sensors, the 1 UART (Universal Asynchronous Receiver-Transmitter) sensor and 1 UART 4G HAT, instead of a mere MCU (Micro-Controller Unit). Although the inclusion of the power unit is not advisable for environments in which high temperatures and intense smoke prevail, an alternative power supply will be sought in a future version in order to better adapt to such difficult conditions. Cooling methods such as fans and heatsinks could also be explored to keep the equipment from thermally throttling. In any case, it is possible to exploit the built-in sensor and the corresponding interface of the Raspberry Pis to inform their users (with appropriate messages) about the temperature levels of the CPUs. Two concerns that must be finally taken into account pertain to the accumulation of fatigue resulting from the transportation of the portable solution in the field over extended periods, despite its relatively light weight, and the adequate training of users to effectively address potential issues that may arise from its use (e.g., inability to connect via 5G and attempt to exploit available mesh-in-the-sky network).

The challenges encountered during the implementation of the portable devices include the following: (a) Both the PM_2.5_ sensors and HAT communicate with the Raspberry Pi using the same data transfer protocol (UART). To prevent data transfer conflicts, we need to create two UART communication channels/interfaces. (b) Despite all sensors being calibrated by the manufacturer, the provided library for the PM_2.5_ sensor is not available in Python. Therefore, we have to implement it from scratch.

#### 3.1.1. The Sensing Unit

There are numerous metrics used in order to evaluate the AQI. The metric we use is the European Air Quality Index (EAQI). This metric is based on the concentrations of five different pollutants, specifically, the concentration of the gasses O_3_, NO_2_ and SO_2_, the particulate matter of diameter 2.5 μm (PM_2.5_), and the particulate matter of diameter 10 μm (PM_10_). This index is used over other indices proposed in the literature since it is more beneficial than others. Table 3 shows the index levels of the AQI and the corresponding values of the pollutants. In order to evaluate the air quality of the environment, we should compute the corresponding index level for each pollutant and compare these values. The maximum index level values indicate the air quality of the environment. Alongside the aforementioned pollutants, another gas sensor is also used. It observes carbon monoxide emissions. This sensor is used for smoke detection and can provide information about the thickness of the smoke and if there is danger of asphyxiation for personnel.

#### 3.1.2. The Communication Unit

As stated previously, the communication unit consists of an extension attached to the processing unit. This extension, in order to be able to provide communication functionalities, comes with two antennas, i.e., a GPS and a 4G antenna. Since the communication is transmitted over the public switched network, a SIM card is required. The SIM card can be a configurable IoT application specific card or a typical market card with sufficient mobile data. The integrated modem is fully programmable and provides capabilities both for data transmission and geospatial information gathering, through the GPS antenna.

The extension is fully capable of transmitting over 4G LTE, like in previous generations. It can transmit a prosperous amount of data in a short amount of time (up to 100 Mbps), given the capability to concurrently transmit data from numerous nodes, given that the amount of data transmitted is small enough. This is useful in emergency cases, where the quality of air worsens faster and faster, or the location of the personnel changes abnormally and they cannot respond to the monitoring department.

#### 3.1.3. The Processing Unit

Raspberry Pi is the primary contestant as a processing unit for such types of applications. This microcomputer offers a variety of applications that can be used to ease the development of such systems. Although it has higher energy consumption compared to other processing units (for example, Arduino and ESP32), it offers more functionalities and ease of software implementation and maintenance. Many sensors offer libraries that are implemented for Python (the basic language that comes with Raspberry Pi). Alongside the software solutions, Raspberry Pi comes with support for all the standard data transmission protocols, allowing multiple connections for these protocols. This restricts the additional hardware equipment (e.g., multiplexers), reducing the size of the node as well as the cost and complexity.

**Figure 4:**
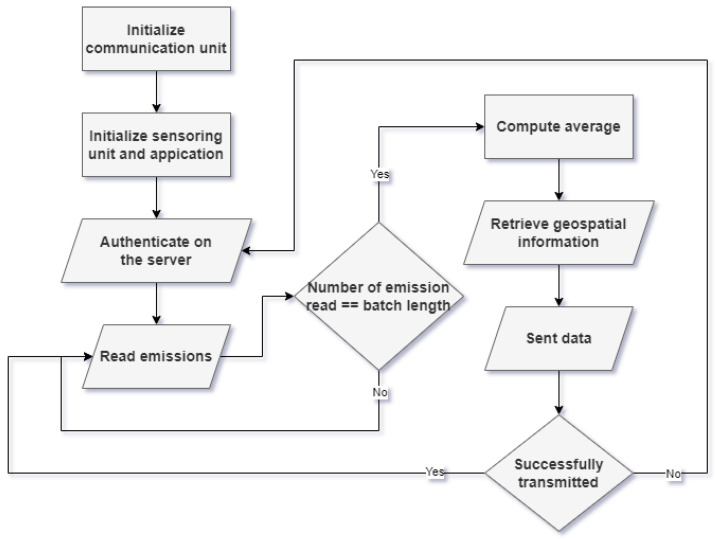
Raspberry Pi algorithm.

The main algorithm that the Raspberry Pi implements is shown in Figure 4. The communication unit initialization is performed with the help of an external command line application, the ModemManager, for enabling the modem for communication, and another application, udhcp for providing a dynamic IP. The emissions that are read from the sensors are temporarily stored in a buffer as a batch. When the buffer has reached a specified amount of records, then the average of the batch is sent to the server. Additionally, geospatial information as well as the unique identifier of the node are attached. The geospatial information is provided by the connection unit. The authentication is performed using unique credentials for each node. The credentials consist of a unique username and a password. Authentication is required since the endpoint communication is performed with a JSON Web Token (JWT) (more details are provided later).

#### 3.1.4. The Power Supply Unit

The power supply of this system consists of a Waveshare Solar Power Manager (B), which functions as battery of a solar panel, although it can be also used individually without the adoption of the solar panel. The battery holds up to 10,000 mAh of power storage, offering adequate power for the running system.

### 3.2. The Stationary Solution

**Figure 5:**
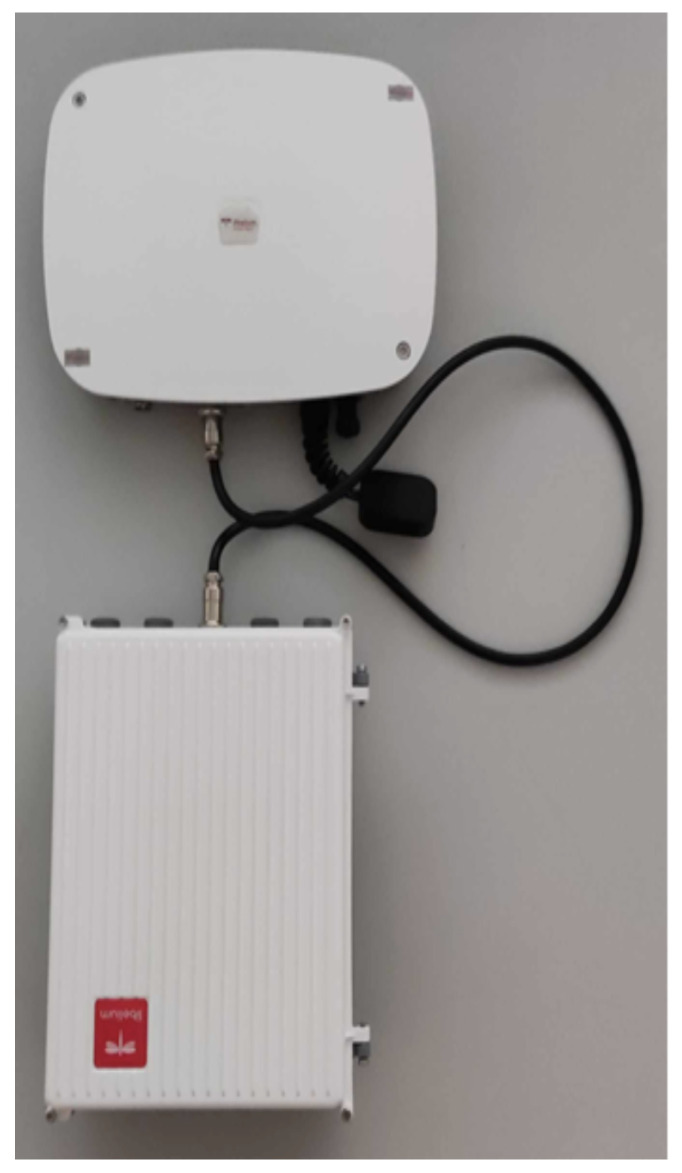
Smart Spot—IoT device for environmental parameter sensing.

We utilize a range of sensors beyond those used by firefighters to monitor wildfire spread in forests. Specifically, we leverage the Libelium Smart Spot model (https://www.libelium.com/iot-products/smart-spot/, accessed on 3 March 2024) (Figure 5). Table 4 presents the specifications of the stationary solution adopted. These ready-made solutions accommodate various IoT applications, including air assessment and environmental aspects. Smart Spot management can be accomplished by using a dedicated platform. The platform encompasses diverse functionalities crucial for effective device management, e.g., monitoring of device status, and connectivity options. A visualization dashboard is also enabled. Two panels are integrated to visually represent the presence of gases (NO_2_ and O_3_) and particulate matter (PM_10_, PM_2.5_, and PM_1_) within the proximity of the deployed smart spot devices.

**Figure 6:**
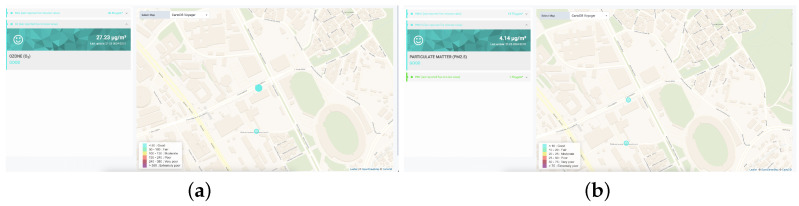
Smart Spot visualization dashboard. (**a**) O_3_. (**b**) PM_2.5_.

Figure 6a,b illustrate pollutant measurements for O_3_ and PM_2.5_ within a specific geographical zone, respectively.

## 4. Data Manipulation and Knowledge Extraction

**Figure 7:**
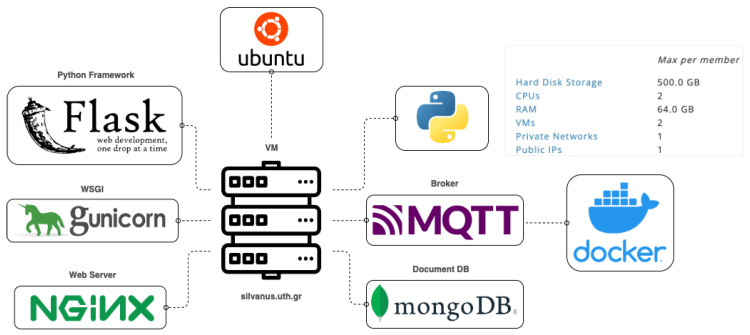
Server characteristics and integrated components.

The continuous operation and seamless request handling of the web application require the utilization of a dedicated web server. Our web server consists of two (2) software solutions, i.e., Gunicorn (https://gunicorn.org/, 4 March 2024) and Nginx (https://www.nginx.com/, accessed on 3 March 2024). The Gunicorn server provides the Python Web Server Gateway Interface (WSGI) established to allow different Python web applications to communicate with the web server program. Typically, a web server program communicates with a backend application using an interface. For back-end applications written in Python, the standard interface for the servers is WSGI. Gunicorn eliminates the need for additional configurations. However, Gunicorn’s limitations in handling network congestion, load balancing, and scalability highlight the necessity for a supplementary solution. For this reason, the Nginx server is implemented to enhance the application’s scalability and tackle security concerns. Figure 7 presents the server’s setup, i.e., the specifications and the integrated software components.

### 4.1. Portable Solution—REST API

Since a Raspberry Pi has limited resources, it cannot efficiently manage extensive data storage without compromising its ability to collect real-time data. Thus, offloading data storage to a more capable system (like a web application server) can ensure that real-time data collection is retained. The data collected from the sensors are stored within a web application. This centralized storage is essential to ensure the data remain in one accessible location for end users. The web application is designed to communicate not only with the Raspberry Pis but also with interested parties seeking access to the data and AQI. It operates on a REST API, facilitating data exchange with other applications. The information obtained from the sensors is stored and organized using a NoSQL database, while access to the API endpoints requires user authentication. The system supports a robust authentication mechanism, employing basic authentication with roles for user management and access control. The RESTful application is implemented in Python, using the Flask framework, and the database management system used to store the data is MongoDB. The RESTful application comprises five endpoints, allowing users to retrieve data directly from the database. Access to these endpoints is restricted solely to verified users possessing specific roles, with each endpoint having distinct role-based access. Table 5 summarizes the deployed endpoints.

Endpoint: insert-data. This endpoint is periodically called from Raspberry Pis to send the data they have collected to the web server. They contain the concentration values of the observed emissions, geographical coordinates, and a unique id. These data are just collected and not processed in the Raspberry Pi. The processing is performed on the server. The data are sent in JSON form as shown in Listing 1. The web application then registers these in two collections, one which contains only the concentrations of the emissions and one which contains the calculated EAQI.
**Listing 1.** /insert endpoint sent a JSON file.{ “Emissions”: {  “CO”: 1.3,  “SO2”: 0.0,  “NO2”: 0.02,  “O3”: 0.01,  “PM1.0”: 3,  “PM2.5”: 4,  “PM10.0”: 7, }, “Pi_id”: 1, “Coordinates”: [38.89709, 22.43608] }


Endpoint: get-latest-data. Using this endpoint, authenticated users can access the database and retrieve the latest chronologically registered data, added from the Raspberry Pi. Users can specify the amount of data they intend to retrieve by inserting it into the URL as an HTTP header argument, formatted as ?emissions=<number>. Data are returned as a list of JSON files in chronological order.

Endpoint: aqi. This endpoint is used to retrieve the most recently registered AQI. It returns a JSON file containing information about the AQI, as shown in Listing 2.

Before delving into more details about the /data-metadata endpoint, we should discuss the Data Ingestion Pipeline (DIP) provided by the SILVANUS project. DIP is a part of the Big Data framework, which serves as a fundamental system integrating components for handling data ingestion, storage, transformation, and processing. It forms the backbone of SILVANUS, facilitating the creation of diverse user software components that can be flexibly deployed across different platforms. Within the Big Data framework, the DIP component is employed to ingest, annotate, and preprocess various data from multiple providers into the storage layer. The DIP directly communicates with the SILVANUS Storage Abstraction Layer (SAL) through a REST API, acting as a mediator between data sources, software components, and the system’s object store. The SAL exposes an abstract POST HTTP endpoint responsible for ingesting all data from the DIP. As part of this process, the DIP sends both the actual data and its associated metadata to the SAL via a specific endpoint. SAL then processes the input, validates the metadata, and confirms that there are no data duplicates. Finally, stored data are forwarded to interested parties via the message bus, implemented by the RabbitMQ (https://www.rabbitmq.com/, accessed on 3 March 2024) message broker.
**Listing 2.** /aqi endpoint retrieved JSON file.{  “AQI”: “Good”,  “position”: {    “Latitude”: 38.89709,    “Longitude”: 22.43608  },  “radius”: 2,  “sensorId”: 0,  “timestamp”: “2024-01-14T10:23:33.675506Z” }


Endpoint: data-metadata. This endpoint periodically posts a multipart HTTP request to the SAL. Two json files are ingested, namely data.json and metadata.json. The former file contains a structured representation of data, specifically related to air quality sensor readings, while the latter file contains metadata information associated with the data.json file.

Endpoint: data-visualize. This endpoint fulfills the need for data visualization in real-time by rendering a dynamic site that plots a bar chart and a line chart. Based on the latest data registered, the line chart consists of five lines, each corresponding to an emission that is used in the calculation of the AQI, while the bar chart presents the actual air-quality levels. Both plots are updated when new emissions observations are registered in the database.

The communication scheme between the component and the web server is as follows: the Raspberry Pi sends a POST request, in order to login and obtain a unique JSON Web Token (JWT). Then, it periodically sends an HTTP POST method request with the data collected from the sensors and the GPS. The data corresponding to sensors are the mean of several previous observations e.g., five observations per request. This translates into reduced bandwidth consumption, maintaining frequent transmissions of crucial information. Whenever the firefighter terminates the operation of the device, the Raspberry Pi sends an HTTP DELETE request to logout and terminate the connection with the web server. It is worth noting that each request involves synchronization (SYN), acknowledgment (ACK) and termination (FIN) messages. Prior to sending the request, a SYN packet is sent from the Raspberry Pi to the server. This triggers a response from the server in the form of a SYN with ACK packet. The Raspberry Pi then transmits the POST request, and the server responds with two ACK packets: one for confirming receipt of the request and another before sending the response. Upon receiving the response, the Raspberry Pi sends the confirmed receipt with an ACK packet, followed by a FIN and ACK packet to end the connection. The server concludes the session by transmitting a FIN ACK packet, and the Raspberry Pi responds with an ACK packet. Table 6 and Table 7 demonstrate the communication protocols and the length of each request and response, respectively.

The total payload for each separate request is summarized herein. The data transmission from the client device to the server has a total length of 2282 bytes for both the request and the response, while the JSON containing the observations is only 199 bytes, and the authentication token is 315 bytes. For the authentication, both the request and the response cost the network capacity 1597 bytes, with the length of the JSON being only 42 bytes. The login-out requires, for both the request and the response, 1437 bytes, with the response JSON having a length of only 22 bytes. The data transmission request is frequently repeated, with the frequency of each repetition being determined by the number of samples required to yield the mean. We arbitrary choose to collect five samples to yield the mean. This approximately results in 2.282 bytes/8 s = 286 bytes/s both for the request and the response. The authentication and logout requests are performed once, so we can calculate the transfer rate for these requests as 1597 bytes/s and 1437 bytes/s correspondingly. Throughout this communication process, it is worth noting that each ACK packet is 66 bytes in length, resulting in a total of 7 packets (66 bytes × 7 = 462 bytes). Similarly, each SYN packet is 74 bytes in length, leading to an additional 148 bytes. In total, the communication process results in a total of 610 bytes being transmitted (462 bytes for ACK packets + 148 bytes for SYN packets).

### 4.2. Stationary Solution—MQTT Broker

Data from this device can be transferred over using the Constrained Application Protocol (https://coap.space/, accessed on 3 March 2024) or the MQTT. Regarding the CoAP protocol, a lightweight machine-to-machine (LwM2M (https://thingsboard.io/docs/reference/lwm2m-api/, accessed on 3 March 2024)) server is already deployed and can be accessed using the URL: coap://homard.hopu.eu:5683. In this work, the MQTT protocol is used, which uses the publish–subscribe model. In such a protocol, multiple clients connect and exchange information with each other through a broker, which is responsible for information distribution. The main MQTT protocol configurations include (a) specifying the server host address (mqtt://silvanus.uth.gr, accessed on 3 March 2024) and port (1883), (b) setting the keep-alive time (120), (c) defining the Last Will and Testament (LWT) topic (lib1attrs) and (d) setting the quality of service (QoS) for published messages (1: at least once). We also select the Mosquitto (https://mosquitto.org/, accessed on 3 March 2024) broker, which implements the MQTT protocol. Mosquitto is designed to work with simple message systems, especially for devices that do not have a lot of power. It is compatible with the latest MQTT protocol, it is open-source, and it offers features like dynamic topics and security through user verification.

**Figure 8:**
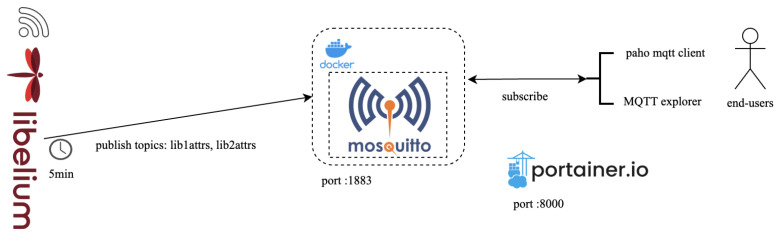
Broker deployment and configuration.

**Figure 9:**
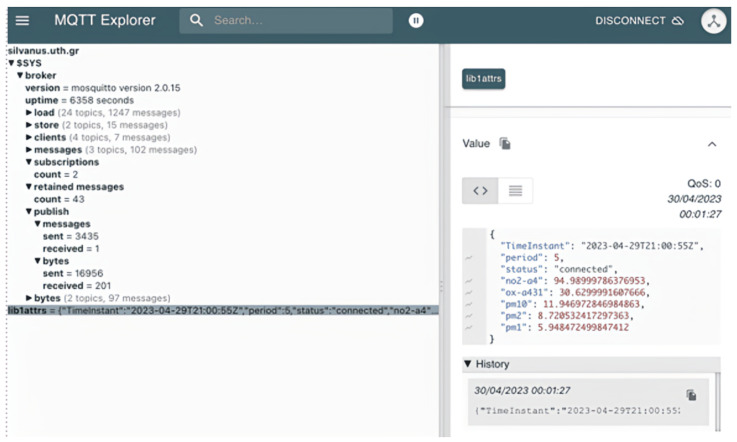
MQTT explorer client.

MQTT broker deployment and exploitation is presented in Figure 8. Initially, we set up a Mosquitto MQTT broker within a Docker container hosted on our virtual machine. Subsequently, we configured each equipment to publish data at five-minute intervals to two specified topics, respectively (lib1attrs and lib2attrs). For end-users/stakeholders who possess an interest in health impact, subscription to our broker is achievable through MQTT clients such as MQTT Explorer or the Paho (https://pypi.org/project/paho-mqtt/, accessed on 3 March 2024) package in Python. Portainer.io is also installed on our VM as a container management tool. The operational procedure of the MQTT Explorer (https://mqtt-explorer.com/, accessed on 3 March 2024) client is illustrated in Figure 9. In this instance, a client is successfully subscribed to the topic “lib1attrs”, and at regular 5 min intervals, it receives a JSON file as depicted in Listing 3.
**Listing 3.** Smart Spot JSON file.{ “TimeInstant”: “2024-01-14T13:23:47Z”, “period”: 5, “status”: “connected”, “no2-a4”: 87.452041562704832, “ox-a431”: 31.826860000506845, “pm10”: 16.162157970869008, “pm2”: 11.228752859573594 “pm1”: 7.8326712017601803 }


In the context of application integration, interested parties can straightforwardly incorporate our system into their operations. This is achieved by initiating HTTP access via our REST API. Detailed information about the API is available on Postman using the following link: https://documenter.getpostman.com/view/29042250/2s9YJZ2iqE (accessed on 3 March 2024). Pollutant measurements obtained from the smart spot device can be accessed through the MQTT protocol as outlined in Section 4.2.

### 4.3. System Scalability and Economic Feasibility

As previously mentioned in the subsections, the solution integrates MQTT and REST API technologies, facilitating scalability. MQTT brokers are capable of managing a considerable quantity of concurrent connections, a crucial aspect for scenarios related to IoT, in which there could exist numerous devices. The adeptness of the broker in overseeing these connections and message transmissions facilitates the efficient scalability of the MQTT protocol. In addition, scalability is an intrinsic characteristic of REST APIs that sets them apart from other types of APIs. The inherent scalability of REST APIs is derived from their stateless architecture. The statelessness of REST APIs refers to the absence of client state preservation by the server across requests. Every interaction between a client and a server in REST APIs necessitates the inclusion of all pertinent information for request comprehension and processing. Managing session data or synchronizing it across numerous servers is not required.

The expenditure associated with the proposed portable solution amounts to around EUR 1000, encompassing value-added tax, with 75% allocated to the expenses related to pollutants sensors. The cost of the stationary solution is similar, including the necessary installation equipment, such as metallic poles and connectors. Available portable equipment can be allocated to discrete members of different firefighting teams operating in an affected area and, combined with the stationary ones mounted on corresponding fire trucks, could effectively assess the surrounding air quality.Each piece of equipment can be distributed to a team of various personnel members, e.g., about 20–30 personnel according to [32].

In terms of maintenance costs and logistical expenses for both solutions, they are almost negligible. The portable solution has minimal weight, resulting in minor logistical costs. As for the stationary solution, it is either fixed to the ground or mounted on a ground vehicle (such as firetrucks), eliminating any associated logistical expenses. Moreover, maintenance expenses are practically non-existent for both solutions since sensors and components are known for their robustness and reliability; they typically consist of solid-state electronics and consume minimal power. Also, both solutions can be troubleshooted remotely.

## 5. Experimental Evaluation

To evaluate this work, two experimental scenarios are conducted. These experiments focus on the behavior of the portable solution in different combustion phases of wildfire, as well as how the values of the concentrations are influenced by the composition of biomass burning. Specifically, a different biomass produces different quantities of the concentrations of these gasses. The AQI is influenced by the quantity of each concentration in a non-linear way. If a specific emission’s value succeeds a threshold, the AQI value is modified, even if the concentrations of the other emissions are intact. Since the gas concentration sensors measure in ppm, conversion between ppm to μg/m^3^ must be applied. The mathematical formula for such a conversion [33] is given below:

$$TLV\;\mathrm{in}\;\mu {g/m}^{3}=\frac{1000\times P\times \mathrm{Molecular}\;\mathrm{Weight}\times TLV\;\mathrm{in}\;\mathrm{ppm}}{62.4\times (273.2+\mathrm{Temperature}\;{}^{\circ}C)}$$

where TLV is the Threshold Limit Value, *P* is the pressure of the gas expressed in mmHg units, and *T* is the temperature expressed in °C units. Under normal circumstances, the value of pressure is 1 atm or 760 mmHg, and the value of temperature is 25 °C. Since all the experiments are performed under normal pressure (atmospheric pressure, 760 mmHg), the above expression is reformulated to the following:

$$TLV\;\mathrm{in}\;\mu {g/m}^{3}=\frac{1000\times 760\times \mathrm{Molecular}\;\mathrm{Weight}\times TLV\;\mathrm{in}\;\mathrm{ppm}}{62.4\times (273.2+\mathrm{Temperature}\;{}^{\circ}C)}$$

**Figure 10:**
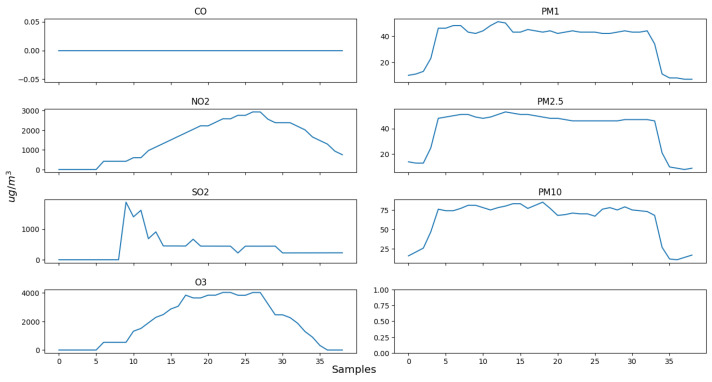
Indoor experiment values for the emissions’ concentrations.

**Figure 11:**
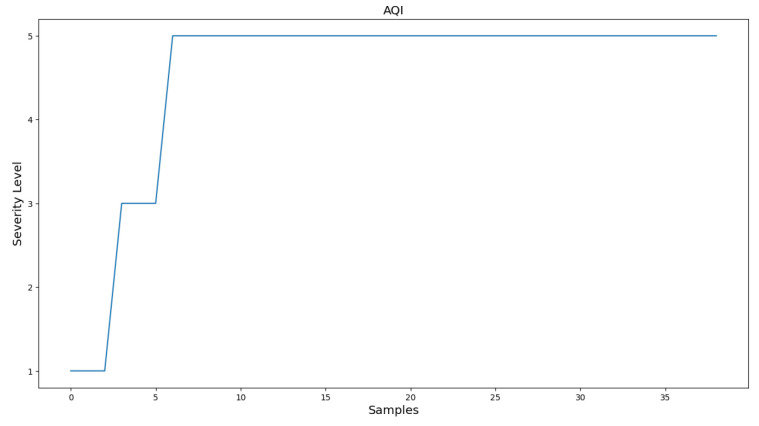
Indoor experiment AQI values.

**Figure 12:**
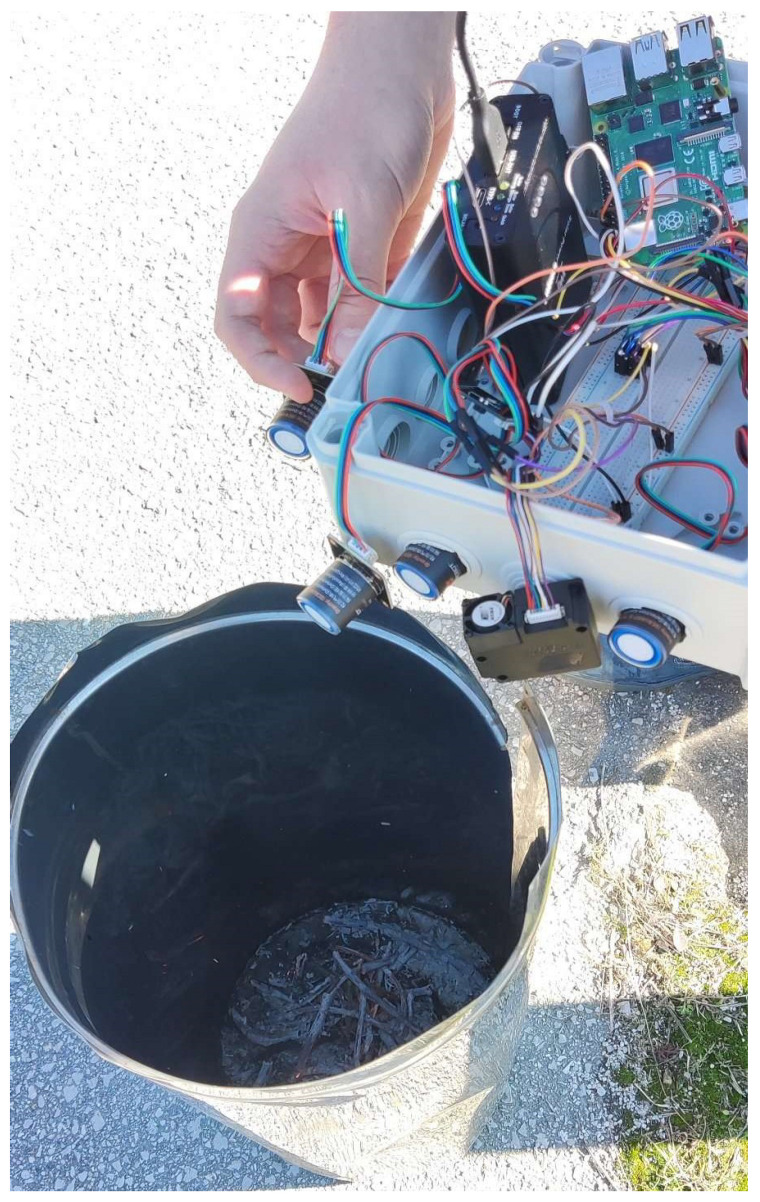
Outdoor experiment settings.

**Figure 13:**
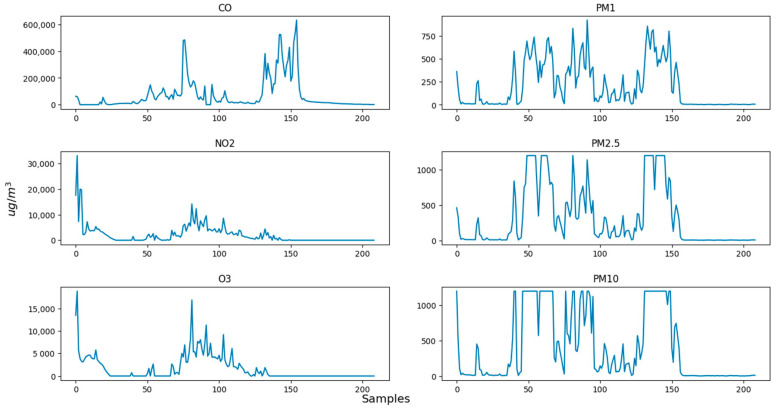
Outdoor experiment values for the emissions’ concentrations.

**Figure 14:**
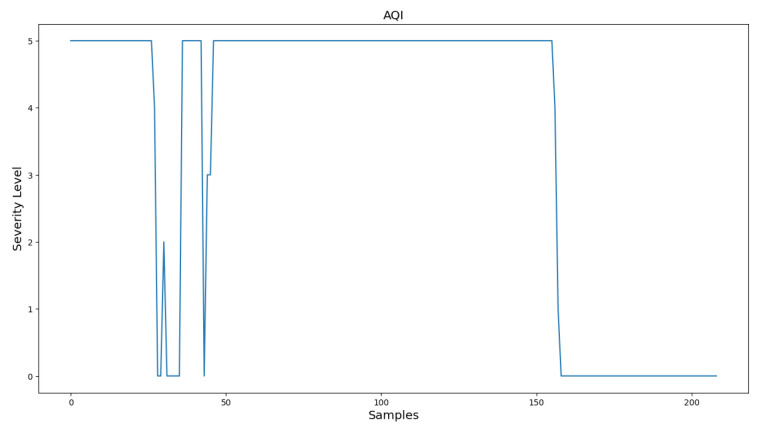
Outdoor experiment AQI values.

It is important to note that not all concentrations of the AQI are important. Due to the potential for elevated levels of SO_2_ in the center of a crowded city [6], which lead to an inaccurate representation of the air quality in those areas, a decision is made to exclude it from the experimental process. For these experiments, one portable solution is utilized for evaluating the measuring accuracy of the portable solution. Figure 10 and Figure 11 present Indoor experiment values for the emissions’ concentrations and the AQI index, respectively. Figure 12, Figure 13 and Figure 14 demonstrate our outdoor experimental setup and outdoor experiment values for the emissions’ concentrations and the AQI index, respectively.

The first experiment process is carried out indoors, where room conditions prevail. The equipment is placed at a distance of about half a meter from a fireplace containing oak logs that are burning, in a position perpendicular to the direction of the smoke. There is no direct contact in this case, and the flames are not very intense. The mean temperature is around 28 °C and the pressure is normal (1 atm or 760 mmHg). Figure 10 illustrates the observations recorded. The sampling period between each reading is one and a half (1.5) seconds. The initial zero values from reading 0 to reading 8 for each pollutant are due to the fact that when the portable system is activated, it is not placed right next to the fire and the smoke. The zero value for the CO is due to the fact that the system is perpendicular and at a relative distance from the smoke. The increasing values of O_3_ and NO_2_ are due to the disposition of the system. The instant increase in the SO_2_ value following a quick decrease is due to the fact that this sensor is purposely exposed closer to the fire for a short period in order to verify the fact that only at a very close distance are significant changes actually achieved regarding its concentration.

On the other hand, the second experiment takes place outdoors in a fully controlled environment taking all the safety measures. The experiment, demonstrated in Figure 12, is conducted on the campus of the University of Thessaly, Lamia, Greece. Strong winds are not present in the area, and the level of relative humidity is almost 42%. The fuel used to perform the experiment consists of dry leaves and small branches of plane trees placed inside a metal bin. It is worth mentioning that the portable IoT device and the built-in gas sensors are directly exposed to the released smoke, and the monitoring of the concentration values of the pollutants of interest is carried out during the flaming combustion stage. The temperature is around 30 °C. Due to the potential for elevated levels of SO_2_ in the center of a crowded city [34], which lead to an inaccurate representation of the air quality in those areas, we exclude SO_2_ concentrations from the second experimental process. This time, when the system is activated, the fire is already burning for an adequate time. The sampling period between each reading is again one and a half (1.5) seconds. The initial slope at the beginning is due to smoke being produced at a lower quantity. This can be concluded from the low CO concentration values, in contrast with the rest of the gas observations, and the low values for a long time span, between observations 15 and 45, where concentrations slowly begin to rise again. Particulate matter decreases slightly earlier but has a spike, possibly due to some dust blown from the air. Some gaps between the specific values are because the light air was blowing the smoke to a different direction, requiring the system to move alongside but, in that time, not facing the smoke. After observation 135, NO_2_ and O_3_ values nullify, while the rest of the pollutants nullify after observation 155. This is because, after observation 155, the smoke and fire are suppressed, and the system is removed from the source of fire and smoke. It is interesting though, that the two gases nullify significantly earlier. This is probably because the burned biomass evaporates completely, leaving only ash and smoke.

Accordingly, the AQI for the second experiment is illustrated in Figure 14. Values 0 to 5 in the y-axis depict step-wise the values of the AQI. ‘Good’ corresponds to 0 and ‘Extremely Poor’ corresponds to 5. We can obviously verify the same result extracted previously. The AQI is constantly high, aside from values 28 to 40, where the fire has been suppressed, and after observation 155, where the system is removed from the source of fire. The difference between that interval and the concentrations interval is due to the spike of the particulate matter. Nevertheless, the overall result verifies what is resulted from the previous figure.

## 6. Conclusions and Future Plans

The generation of harmful emissions as a consequence of the combustion of biomass necessitates the development of systems that are capable of enhancing the safeguarding of the health and safety of individuals (e.g., firefighters and emergency responders) within an affected area. In this paper, we propose an integrated system whose main objective is to monitor the air quality of an area where a fire incident occurs and seamlessly inform stakeholders in order to mitigate detrimental health implications. Adopting a network of IoT devices, portable and stationary, equipped with multiple gas and particle sensors, the proposed system manages to monitor the geospatial progression of the pollutants concentrations. These field observations are wirelessly delivered to a remote server which is responsible for performing, adopting the methodology of EAQI, the air quality characterization, and then sharing through appropriate RESTful APIs and visualizing this inferred information to interested parties. The proposed system could be a module of an integrated fire management decision support system.

The presentation of this solution over the course of a tabletop exercise took place in Evia on 31 October 2023, and received very positive feedback from the stakeholders (firefighters, first responders, civil protection authorities, and policymakers) who participated. They expressed significant interest in portable and stationary IoT devices designed for monitoring the quality of ambient air and estimating the health impacts on firefighters resulting from the occurrence of fire. It was appreciated that part of the equipment was portable, as firefighters could attach it to their trucks and carry it about with ease. Nonetheless, there exist a number of challenges necessitating attention to facilitate extensive implementation. Issues such as the number of devices needed, the assurance of hardware security and the appropriate stakeholders training have all been identified as areas of concern. Our future plans encompass the incorporation of real-time biometric data pertaining to the firefighters and nearby citizens, thereby empowering civil protection authorities to possess a more comprehensive picture of their well-being. Subsequently, the improved system will be subjected to experimental assessment in real-world situations. The process of evaluating the system in the field would provide us with the capability to not only monitor and record real-time data about pollutant concentrations but also to control various aspects (such as energy autonomy and communication assurance) that ensure its optimal functioning in challenging environmental conditions. An extensive pilot testing plan is underway that includes the adoption of this solution in the field under various fire incident scenarios. Another promising direction is the implementation of machine learning algorithms for the predictive analysis of air quality during wildfires. The use of predictive analysis during the wildfire provides valuable insight for wider wildfire-monitoring decision support systems, as well as assisting the guidance of front line wildfire firefighters. Additionally, the incorporation of federated learning could provide a sufficient layer of security and network overhead reduction. In a scenario where malicious attackers access the channel and manipulate the transmitted values of the emissions, wildfire firefighters could be misguided and potentially harmed. Subsequently, if the learning process is accomplished through capable enough devices that are nearby with each other, the overhead would be significantly reduced. These advancements have the potential to significantly improve wildfire management and the protection of wildfire firefighters.

## Figures and Tables

**Table 1:** Firefighter-monitoring solutions.

Reference	Values	Position	Network Infrastructure	Processing Unit
[10]	Body Movement, Heart Rate, Temperature, Smoke, GPS	Wearable (Bracelet)	LAN-Wi-Fi 5G	-
[11]	Heart Rate, Temperature, Smoke Gases Concentration	Outdoor	Bluetooth 3.0SPP,	-
[15]	Body Movement, Blood Volume Pressure, Echocardiogram, Electrothermal Activity, Electroencephalogram, Electromyogram, Respiration, Temperature	In-body, On-body, Off-body, Mixed	Wearable Sensor Network, Short Range Radio Frequency Standards	-
[12]	Heart Rate, Temperature, Body Movement	Wearable (Helmet)	LoRa, Bluetooth Low Energy	ESP32
[9]	Heart Rate, Body Temperature, Air Pressure, Beidou	Wearable (Watch)	LoRa, 4G	-
[20]	Flame Detection, Flammable Gas Concentration, Human Detection, Temperature, Humidity	Robot	Wi-Fi	ESP32
[16]	RFID Epidermal Temperature	Epidermal, Suit Integrated	Wireless Body Sensor Network, RFID	None used
[7]	Galvanic Skin Response, Heart Rate, Temperature, Humidity, Gas Concentration	Wearable (Gloves)	Wi-Fi, Xbee, Zigbee, esp8266, MQTT	Arduino
[8]	-	Wearable (Waist Belt)	Mobile Ad Hoc Network	-
[19]	Electrocardiogram	Suit integrated	Bluetooth Low Energy	Custom System
[18]	CO concentration, Pulse Rate, Posture Detection, Temperature, Warning Buzzer	Suit integrated	Any transmitter operating at ISM 2.4GH	Arduino
[17]	Heart Rate, Movement, Gas Concentration, Temperature, Relative Humidity, GPS	Suit integrated, Commander Control Unit Compontent	Body Area Network Wide Area Network textile bus system Bluetooth version 4	Arrietta G25
[14]	Accelerometer	Intelligent Garment	Xbee	Arduino ATmeg32U4
[13]	Temperature	Wearable	Wireless Body Area Network, 6LoWPAN	CC2650 Multistandard Wireless MCU, Cortex-M3, Cortex-M0

**Table 2:** Environmental monitoring solutions.

Reference	Pollutants	Processing Unit	Network Infrastructure
[25]	CO, CO_2_, SO_2_, NO_2_, PM_2.5_	Arduino Uno	LoRaWAN
[22]	PM_1.0_, PM_2.5_, PM_10.0_	Node MCU	Wi-Fi
[27]	PM_2.5_, PM_10.0_, CO, NO_2_, O_3_	BMD-340 System On a Module	BLE, Wi-Fi
[21]	O_3_, NO_2_, SO_2_, CO	Intel Edison	Wi-Fi
[24]	PM_2.5_, PM_10.0_, NO_2_, O_3_	PIC32MM0256GPM048	BLE
[29]	NO_2_, SO_2_, O_3_, PM_10.0_, PM_2.5_, CO_2_, VOC	ESP32	Wi-Fi
[23]	PM_10_, PM_2.5_, SO_2_, NO_2_, CO, O_3_, CO_2_, NO, CH_4_, HC, H_2_	Waspmote Gas Sensor Board 3.0	LoRaWAN
[26]	-	Raspberry 3B+	Wi-Fi
[28]	CO, PM_2.5_	STM32F103C8T6	Wi-Fi

**Table 3:** European Air Quality Index (measured in μg/m^3^).

	Good	Fair	Moderate	Poor	Very Poor	Extremely Poor
PM_2.5_	0–10	10–20	20–25	25–50	50–75	75–800
PM_10_	0–20	20–40	40–50	50–100	100–150	150–1200
NO_2_	0–40	40–90	90–120	120–230	230–340	340–1000
O_3_	0–50	50–100	100–130	130–240	240–380	380–800
SO_2_	0–100	100–200	200–350	350–500	500–750	750–1250

**Table 4:** Libelium Smart Spot specifications.

OS	FreeRTOS
CPU	Dual Core 240 MHz
RAM	16 MB
Connectivity	Wi-Fi, NB-IoT
Remote Control	Homard Platform
Energy Consumption	180–300 mA Active
Voltage	5V
Size	300 mm × 200 mm × 36.7 mm
Weight	1.8 kg
Gas Sensors	O_3_, NO_2_
Particle Sensors	PM_1.0_, PM_2.5_, PM_10_
Wind Parameters	Temperature, Humidity, Pressure

**Table 5:** Endpoints description.

Endpoint	HTTP Method	Description	Parameters
/insert-data	POST	Send data to the server.	JSON file Authentication Credentials
/get-latest-data	GET	Get the latest added data.	Number of data Authentication Credentials
/aqi	GET	Get the most recent AQI measurement.	Authentication Credentials
/data-metadata	POST	Send data to SILVANUS Cloud	SILVANUS Credentials
/data-visualization	GET	Emissions and AQI visualization.	Authentication Credentials

**Table 6:** Requests.

Headers	Data Transmission	Authentication	Logout
Method	POST	POST	DELETE
Version	HTTP/1.1	HTTP/1.1	HTTP/1.1
URI	/insert-data	/auth/login	/auth/logout
Host	silvanus.uth.gr	silvanus.uth.gr	silvanus.uth.gr
User-Agent	python-request/2.25.1	python-request/2.25.1	python-request/2.25.1
Accept-Encoding	gzip, deflate	gzip, deflate	gzip, deflate
Authorization	<JWT>	-	<JWT>
Accept	*/*	*/*	*/*
Connection	keep-alive	keep-alive	keep-alive
Content-Length	199	42	0
Content-Type	application/json	application/json	-

**Table 7:** Responses.

Headers	Data Transmission	Authentication	Logout
Response Version	HTTP/1.1	HTTP/1.1	HTTP/1.1
Status Code	200	200	200
Response Phrase	OK	OK	OK
Server	nginx/1.10.3 (Ubuntu)	nginx/1.10.3 (Ubuntu)	nginx/1.10.3 (Ubuntu)
Date	<Datetime_Of_Response>	<Datetime_Of_Response>	<Datetime_Of_Response>
Content-Type	text/html; charset=utf-8	application/json	application/json
Content-Length	2	42	22
Connection	keep-alive	keep-alive	keep-alive

## Data Availability

Data can be retrieved using the provided REST API or the MQTT broker.

## Abbreviations

The following abbreviations are used in this manuscript:

CO	Carbon Monoxide
CO_2_	Carbon Dioxide
O_3_	Ozone
NO_2_	Nitrogen Dioxide
SO_2_	Sulfur Dioxide
IoT	Internet Of Things
AQI	Air Quality Index
EAQI	European Air Quality Index
PM_2.5_	Particulate Matter of diameter 2.5 μm
PM_10_	Particulate Matter of diameter 10 μm
JWT	JSON Web Token
WSGI	Web Server Gateway Interface
DIP	Data Ingestion Pipeline
SAL	Storage Abstraction Layer
I2C	Inter-Integrated Circuit
UART	Universal Asynchronous Receiver/Transmitter
UAV	Unmanned Aerial Vehicle
UGV	Unmanned Ground Vehicle
MCU	Micro-Controller Unit
